# Mediating Effects of Sarcopenia on Frailty, Cognition, and IADL Disability in Community-Dwelling Elders

**DOI:** 10.1093/geroni/igaf122.1947

**Published:** 2025-12-31

**Authors:** Bei Cheng, Ping He, Jing Ge, Wenhan Li, Aihong Liu, Ling Li, Tangmeng Guo, Kemeng Zhang

**Affiliations:** Union Hospital, Tongji Medical College, Huazhong University of Science and Technology, Wuhan, Hubei, China (People’s Republic); Union Hospital, Tongji Medical College, Huazhong University of Science and Technology, Wuhan, Hubei, China (People’s Republic); Union Hospital, Tongji Medical College, Huazhong University of Science and Technology, Wuhan, Hubei, China (People’s Republic); Union Hospital, Tongji Medical College, Huazhong University of Science and Technology, Wuhan, Hubei, China (People’s Republic); Union Hospital, Tongji Medical College, Huazhong University of Science and Technology, Wuhan, Hubei, China (People’s Republic); Union Hospital, Tongji Medical College, Huazhong University of Science and Technology, Wuhan, Hubei, China (People’s Republic); Union Hospital, Tongji Medical College, Huazhong University of Science and Technology, Wuhan, Hubei, China (People’s Republic); Union Hospital, Tongji Medical College, Huazhong University of Science and Technology, Wuhan, Hubei, China (People’s Republic)

## Abstract

**Background:**

Frailty, cognitive decline, and disability are prevalent among older adults and significantly impact the health of community-dwelling elderly individuals. This study investigates the associations between frailty, cognition and IADL disability and explores mediating factors affecting frailty in this population.

**Methods:**

This cross-sectional study included 571 older adults (aged >65 years) from the Junshan community. Frailty (Fried phenotype), IADL disability (IADL Scale), and cognition (Mini-Cog) were assessed alongside sarcopenia (skeletal muscle mass index, SMI). Spearman correlation analysis was conducted to examine the associations between frailty severity and various factors, multiple single-mediation models were constructed to explore the mediating roles of sarcopenia in the relationships.

**Results:**

Age, BMI, SMI, IADL Score and cognitive function were significantly related with frailty. Mediation analysis revealed that sarcopenia played significant mediating roles in the effect of frailty on cognitive function (β=-0.4247, p < 0.0001) and the reciprocal effect of cognitive function on frailty (β=-0.5818, p < 0.0001). Similar mediation effects were observed in the relationship between frailty and IADL disability (β=-0.6256, p < 0.0001) and the reciprocal effect of IADL disability on frailty (β=-0.6170, p < 0.0001).

**Conclusions:**

The findings indicate that sarcopenia plays a crucial mediating role in the interactions between frailty, cognitive decline, and IADL disability. Implementing comprehensive interventions that focus on enhancing muscle function may help disrupt this vicious cycle, thereby slowing the progression of frailty, preserving cognitive function, and mitigating disability among community-dwelling older adults.

